# Insight in the transcriptome data of hairy root disease-causing bacterium-*Agrobacterium rhizogenes*

**DOI:** 10.1016/j.dib.2020.105910

**Published:** 2020-06-21

**Authors:** Akhilesh Yadav, Hariom Verma, Waquar Akhter Ansari, Asha Lata Singh, Major Singh

**Affiliations:** a Department of Botany, Banaras Hindu University, Varanasi-221005, India; b Crop Improvement Division, Indian Institute of Vegetable Research, Varanasi-221305, India

**Keywords:** *Agrobacterium rhizogenes*, Annotation, Illumina nextseq, Transcriptome, RNA-seq

## Abstract

*Agrobacterium rhizogenes* induce the production of the hairy root through the transformation of plant genomes. In this article, we executed the transcriptome of *A. rhizogenes* through RNA-sequencing. RNA-sequencing of *A. rhizogenes* generated a total of 2.6 Gb raw data with a 75 bp paired-end sequence. The raw data has been submitted to the SRA database of NCBI with accession number SRR5641651. Reads were generated 2946 unigenes and all unigenes were annotated in the database. The length of transcripts ranged from 90 to 6369 bp, with a median transcript length of 968. The transcripts were annotated through the number of databases to obtain information about SSRs, SNPs, Gene Ontology, Transcription factors, and pathways analysis .

**Specification table**

**Table:** 

Subject	Biology
Specific Subject Area	Agricultural Microbiology
Type of data	Transcriptome assembly, raw sequences, Tables, Figures
How data were acquired	High-throughput RNA sequencing using Illumina NextSeq 500
Data format	Raw reads (fastq), analyzed
Parameters for data collection	RNA-Seq data of *Agrobacterium rhizogenes* under control condition were analysed.
Description of data collection	Total RNA was extracted using Quick-RNA Miniprep plus kit, as per the manufacturer's instruction. The RNA-Seq paired and sequencing library was prepared from the RNA sample using Illumine TruSeq stranded mRNA sample preparation kit, according to the manufacture's instruction. The PE Illumine library was sequenced by Illumina NextSeq 500.
Data source location	A sample of *Agrobacterium rhizogenes* was collected from the agricultural filed of Varanasi, India.
Data accessibility	The data are available as a Bio Project hosted at NCBI
	Repository name: Bio Project, NCBI Data identification number: PRJNA388804
	Direct URL to data: https://www.ncbi.nlm.nih.gov/sra/SRX2879936[accn]

**Value of the data**

• The *Agrobacterium rhizogenes* transcriptome can be utilized as a reference for RNAseq data expression study. With the great agricultural significance of *Agrobacterium rhizogenes* these data will provide the path to plan future research programs targeting this bacterium.
• The RNA-seq and assembled transcriptome datasets make available real expression evidence, a researcher working on *A. rhizogenes* may benefit from these data to understand the complexity.
• This transcriptomics data might be useful to understand molecular processes in this bacterium and for comparative transcriptome analyses.

## 1 Data

**Fig. 1:**
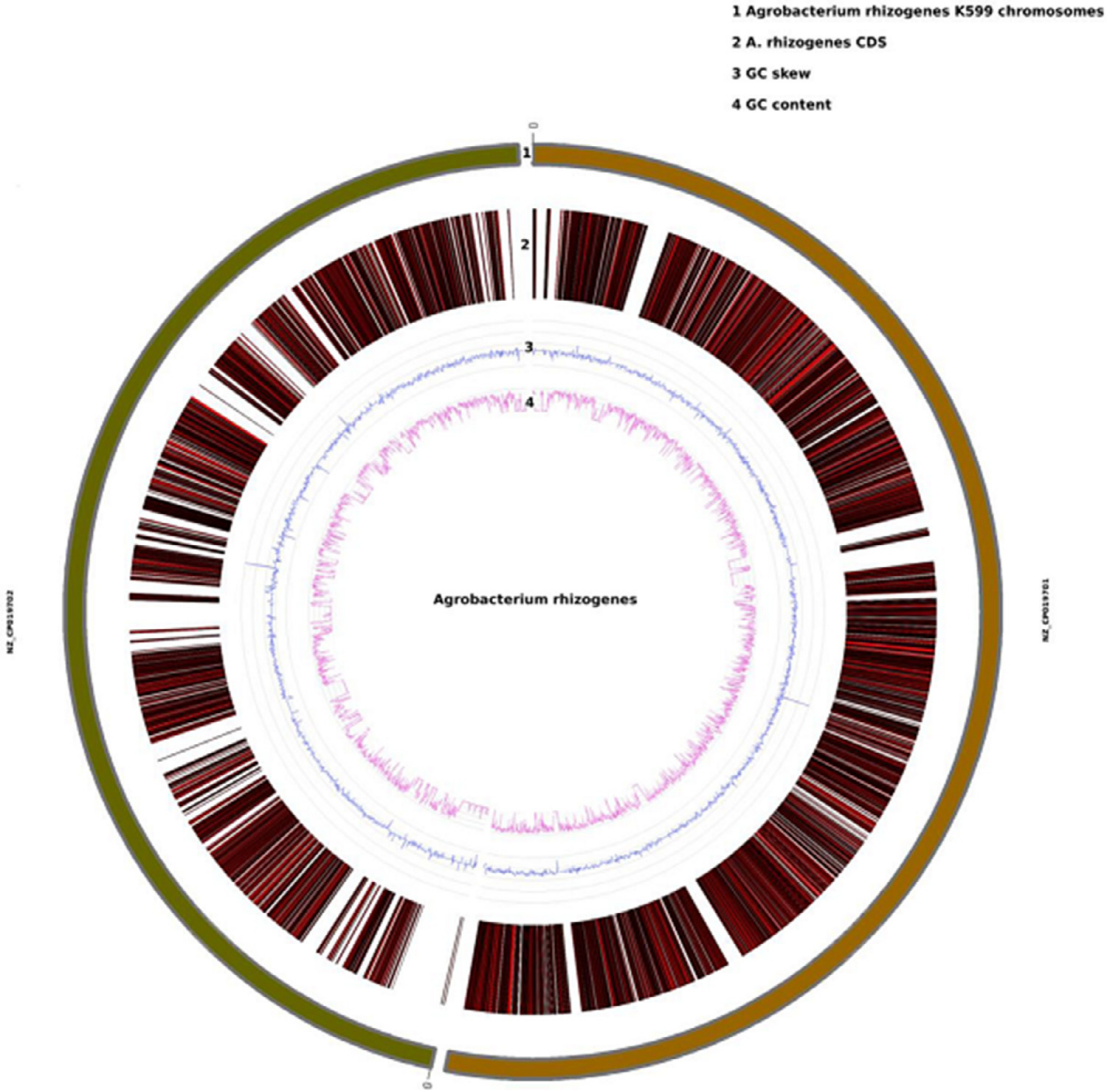
Circular representation of *Agrobacterium rhizogenes* transcriptome*.*

RNA-sequencing of *A. rhizogenes* generated 2.6 Gb raw data with a 75 bp paired-end sequence and were mapped with the reference sequence using Tophat [1]. Particulars of raw reads generated, and gene information is provided in Table 1. The total GC content 37.86% were analyzed and this provides insights into thermostability, gene regulation, and evolution (Table 1). The transcripts were annotated through databases like GO, KEGG, KOG, etc. The functional annotation of genes was carried out against the curated KEGG GENES database using KAAS (KEGG automation server; https://www.genome.jp/kegg/ko.html) [2]. The KEGG orthology database of *alpha-proteobacteria* such as *Rhizobium, Agrobacterium, Sinorhizobium,* and *Mesorhizobium* was used as the reference for pathway mapping. A total of 2946 genes of *Agrobacterium rhizogenes* were used for the pathway analysis. These genes were classified into 24 functional pathway categories which enriched 1842 genes of *Agrobacterium rhizogenes* in KEGG DB respectively (Table 2). The genes identified in *Agrobacterium rhizogenes* along with GC content and GC skew were circularly visualized via the online web-server Circos plotting tool (ClicO FS) for the distribution of genes [3]. The reference chromosome is represented in track 1. All the 2946 genes of *Agrobacterium rhizogenes* sample are highlighted in track 2. GC skew and GC content estimated over a sliding window of 1000 bp are displayed in track 3 and 4 respectively (Fig. 1). The raw data has been submitted to the SRA database (http://www.ncbi.nlm.nih.gov/sra) with accession number SRR5641651.

**Table 1:** Summary of sequencing reads.

Sample name	Agrobacterium rhizogenes
Total number of bases	2625,536,905
Total number of filtered read	17,422,715
Read (in GB)	2.6
Mapping%	98.5
Coverage%	63.15
GC%	37.86
Number of genes	2946
Maximum length of gene	6369
Minimum length of gene	968
Mean of gene length	90

**Table 2:** KEGG pathway classification.

**Pathways**	**No of genes**
**Metabolism**	
Carbohydrate metabolism	189
Energy metabolism	132
Lipid metabolism	63
Nucleotide metabolism	92
Amino acid metabolism	184
Metabolism of other amino acid	51
Glycan biosynthesis and metabolism	30
Metabolism of cofactors and vitamins	108
Metabolism of terpenoids and polyketides	16
Biosynthesis of other secondary metabolism	27
Xenobiotics biodegradation and metabolism	46
Other metabolic pathways	166
**Genetic Information processing**	
Transcription	4
Translation	74
Folding, sorting and degradation	29
Replication and repair	38
**Environmental information processing**	
Membrane transport	253
Signal transduction	90
Signaling molecules and interaction	1
**Cellular Process**	
Transport and catabolism	6
Cell growth and death	36
Cellular community- prokaryotes	154
Cell motility	51
**Organismal Systems**	
Environmental adaptation	2

**Fig. 2:**
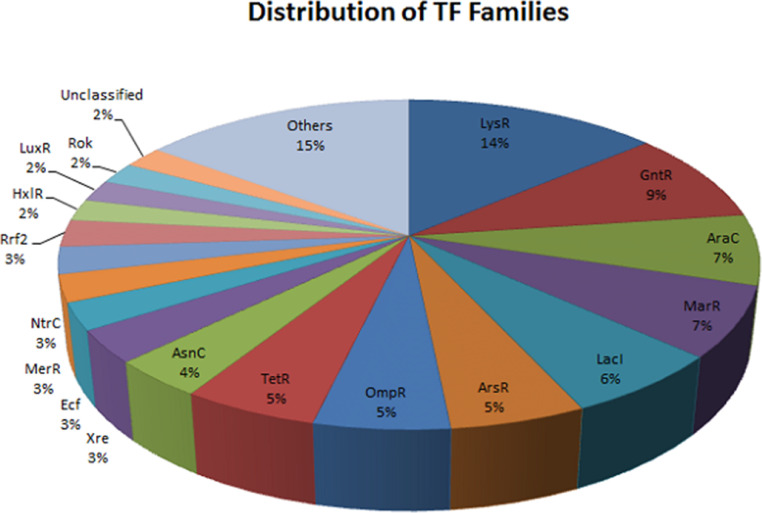
Distribution of different transcription factors (TFs) families in *Agrobacterium rhizogenes.*

A total of 44 predicted SSRs were identified using Microsatellite Identification Tool (MISA v1.0) from the transcript (Table 3) [4]. Transcripts were also used to identify the SNPs and total numbers of 365 SNPs were identified in the sample (Table 3). Transcription factor associated genes (TFs) have been identified based on sequences homology search via BLASTn to 429 known TFs of *Agrobacterium tumefaciens* strain C58 deposited in the P2TF database [5,6]. Out of 2946 genes in *Agrobacterium rhizogenes*, 187 are associated with transcription factors (Table 4; Fig. 2).

**Table 3:** Statistics of SSRs and SNPs identification.

**Particular**	**Filtered**
Total number of identified SSRs	44
Total number of predicted SNPs	365 (3-Heterozygous; 362-Homozygous)

**Table 4:** Summary of different transcription factors (TFs) type.

Type of transcription factor	Genes
Transcription regulator (TR)	100
One-component system (OCS)	55
Response regulator (RR)	17
Sigma factor (SF)	11
Other DNA-binding protein (ODP)	5

**Fig. 3:**
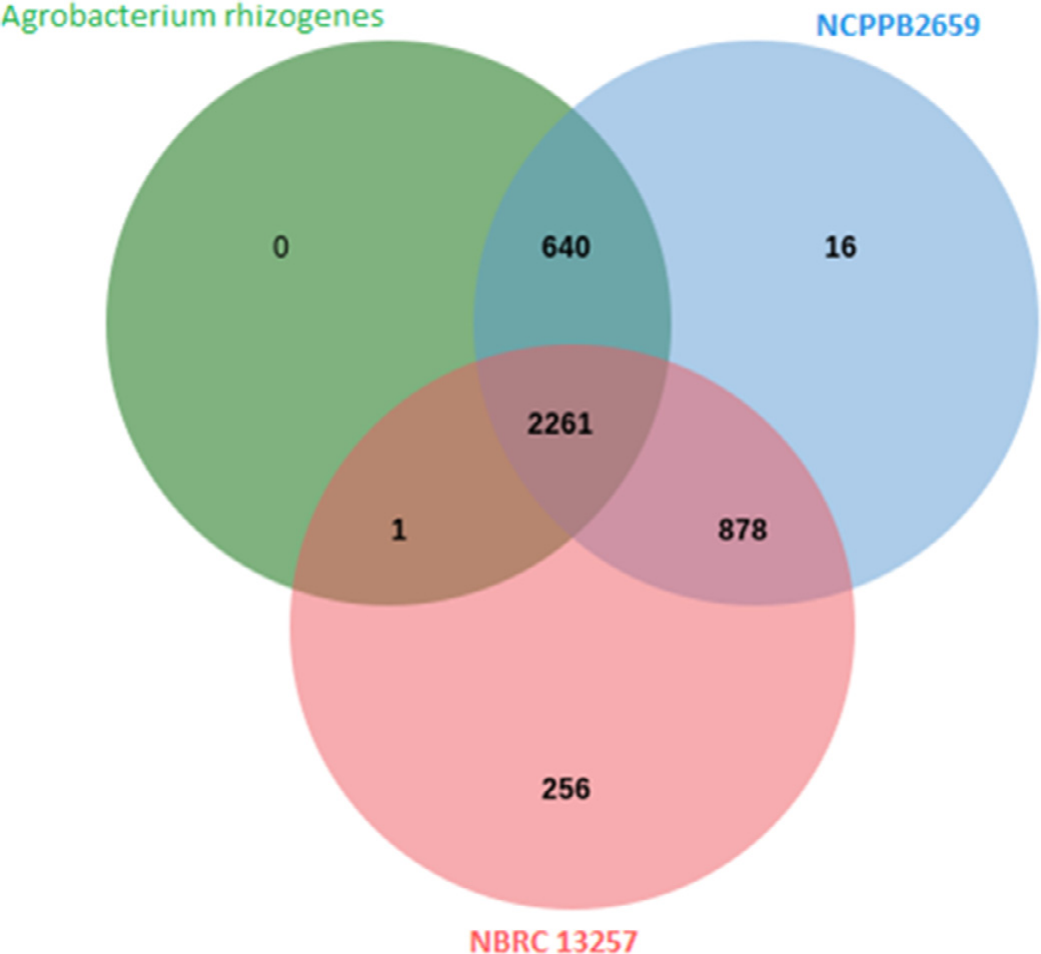
Venn diagram of orthologous clusters. 2261 clusters are found to be common between all the 3 organisms.

Orthologous genes were identified using the Orthovenn program [7]. Orthologs of *Agrobacterium rhizogenes* were identified in the *Rhizobium rhizogenes* strain NBRC 13,257 and *Agrobacterium rhizogenes* strain NCPPB2659 (Fig. 3). Gene ontology (GO) analysis for biological process, cellular component, and molecular function of the protein which is involved in the 2261 and 640 clusters are mentioned in Table 5. The present transcriptomic profiling of *A. rhizogenes*, might be useful for comparative transcriptome analyses and understand the pathway of different biological processes as well as for the development of different biological markers such as SSR, SNP, etc.

**Table 5:** Gene Ontology (GO) summary.

Particular	GO of 2261 ortholog common cluster	GO of 640 ortholog cluster
Biological process	102	76
Cellular component	50	25
Molecular function	35	22

## 2 Experimental design, materials, and methods

### 2.1 Bacterial strain and growth condition

Bacterium *A. rhizogenes* was used to perform this experiment and isolated from the agricultural research field of Varanasi, India (25.28°N 82.96°E). The *A. rhizogenes* was grown in tryptone yeast (TY) broth medium at 28 °C in a New Brunswick Scientific (Edison, NJ, USA) Innova Model 4230 refrigerated incubator shaker at 180 rpm. At the end of the exponential phase, cells were harvested to isolate RNA.

### 2.2 RNA extraction, library preparation, and sequencing

Total RNA was extracted using Quick-RNA Miniprep plus kit (ZYMO Research, California, USA) as per the manufacturer's instruction. RNA concentration, purity, and integrity were assessed using Nanodrop and 1% agarose gel, respectively. Bacterial mRNA was enriched from the total RNA using the MICROBExpress Kit (Ambion, California, USA) as per manufacture's instruction. The RNA-Seq paired and sequencing library was prepared from the RNA sample using Illumine TruSeq stranded mRNA sample preparation kit (Illumina, California, USA), according to the manufacture's instruction. The PCR enriched library was analyzed in a 4200 tape station system (Agilent Technology, California, USA). The PE Illumine library was sequenced by Illumina NextSeq 500 and 75 bp paired-end raw reads were generated.

The raw sequenced data were processed to obtain high-quality clean reads using Trimmomatic V0.35 to remove adapter sequences, ambiguous reads (reads with unknown nucleotides “N” larger than 5%), and low-quality sequences (read with more than 10% quality threshold (QV) <20 Phred score) [8]. A minimum threshold length of 50 bp has been imposed during trimming. The high quality (QV>20), paired-end reads were used for reference-based read mapping with *Agrobacterium rhizogenes* strain K599 using TopHat [1].

## Declaration of Competing Interest

The authors declare no conflict of interest. The author Dr. Hariom Verma is currently working as an assistant professor in the Department of Botany, B.R.D. Government Degree College, Sonbhadra, India.

## Appendix Supplementary materials

Image, application 1
